# Sour Lake, Texas

**Published:** 1900-04

**Authors:** C. H. Wilkinson

**Affiliations:** Health Physician, Galveston, Texas


### SOUR LAKE, TEXAS.

### ITS WONDERFUL PROPERTIES FOR THE RELIEF OF THOSE AFFLICTED WITH DISEASES OF THE DIGESTIVE APPARATUS, AND FOR THE CURE OF ECZEMA, AND SIMILAR CUTANEOUS AFFECTIONS.—A "LEMONADE WELL."

BY C. H. WILKINSON, M.D.,
Health Physician, Galveston, Texas.

Among the many wonderful watering-places in the great Lone Star State, there is none perhaps that has acquired more notoriety, certainly there is none that has attained more deserved popularity, by reason of its intrinsic medicinal virtues than Sour Lake, in Hardin county, Texas.

Situated in the southeastern portion of this great State, on the verge of the "Big Thicket," and buried in the bosom of majestic oaks and pines, this famous retreat for invalids may be truly styled the rural watering-place of America.

Years ago, before the pale face of civilization appeared upon the Western continent, this natural sanitarium must have been known to the American Indian, as legends are now and then gathered, telling of the awe and veneration entertained by the aborigines for the magic power supposed to be mingled with its waters.

Indeed, there is evidence to support the view that this very spot was the objective point of Ponce de Leon when he landed on the shore of Florida in search for the Fountain of Perpetual Youth. Be this as it may, the antiquity of Sour Lake is well established, for no person is so old in Texas to-day, who is not able to give his atom of experience with its great medicinal products.

Sour Lake is a curiosity of nature. Its name might possibly suggest an insignificant body of water entirely surrounded by land. The lake itself is but a fraction of the properties collected there. Nineteen springs, or wells, of differing chemical variety, some only separated from each other a few feet or yards at most, are scattered about the place encircling the lake, all pregnant with minerals and bubbling with gas.

Chemically speaking, the general character of the waters, both of the lake and of the wells, is that of acid sulphate, yet within a few feet of the sourest well is found another marked by antacid. Again, a well of natural tar and one of boiling gas are added to the group. Free sulphuric acid exists in several of these wells, as set forth in an analysis made by Professor Mallet many years ago, an exceedingly rare occurrence in nature, and natural lemonade can be obtained at any time of the year by simply dipping for it. It will be seen by the following formulas that a greater variety of natural waters are collected here on this noted spot of a few acres than upon any other space of its size, perhaps, in the whole of America. This analysis was made by Regis Chauvenet & Bro., Analytical Chemists, St. Louis, Missouri, and is as follows :

No. 1.—Grains of solid salts, per gallon........................17.69

Consisting of the following salts:

Carbonate of lime (quite heavy),
Carbonate of magnesia (quite heavy),
Chloride of iron,
Chloride of potassium,
Chloride of sodium (faint).

The water is remarkably free from sulphates, and its residue showed no traces of organic matter. When received, it had no odor, no traces of sulphuretted hydrogen or other free gases could be discovered.

No. 2.—Grains of solid matter, per gallon........................59.79

Consisting of the following salts:

Sulphate of alumina (strong),
Sulphate of iron (strong),

Sulphate of lime (faint),
Sulphate of magnesia (faint),
Chloride of iron,
Sesquioxide of iron (floating),
Organic matter.

The water is clear, without free sulphur, or any other gases that are perceptible. It has the distinctly acid twang of the soluble sulphates of iron and alumina.

No. 3.—Grains of solid matter per gallon..........................67.46
Consisting of the following salts:
Sulphate of alumina (strong),
Sulphate of iron (strong),
Sulphate of lime,
Chloride of iron,
Sesquioxide of iron (floating).
Free sulphur (faint),
Organic matter.

Has a decidedly acid taste, similar to that of No. 2, is less clear and shows greater tendency to precipitate free sulphur.

No. 4.—Grains of solid matter per gallon..........................22.20
Consisting of the following salts:
Sulphate of lime (strong),
Sulphate of iron,
Chloride of iron (faint),
Free sulphur,
Organic matter.

This water shows quite a free floating bituminous or tarry matter, and has a strong odor of petroleum or naphtha, while its residues are sticky with the organic tars and tarry compounds.

No. 5.—Grains of solid matter per gallon..........................20.76
Consisting of the following salts:
Carbonate of lime,
Carbonate of magnesia,
Sulphate of lime,
Sulphate of magnesia,
Chloride of iron (faint),
Silica (in solution),
Chloride of potassium,
Chloride of sodium,
Free sulphur,
Sulphuretted hydrogen gas (strong),
Organic matter.

This is a strong sulphur water, giving off its sulphuretted hydrogen freely, even after transportation, and throwing down free sulphur quite largely.

No. 6.—Grains of solid matter per gallon.............................68.44

Consisting of the following salts:

Sulphate of alumina (strong),
Sulphate of lime,
Sulphate of iron (strong),
Sulphate of magnesia (faint),
Soluble silica,
Chloride of iron,
Free sulphur,
Organic matter.

This water also shows an oily coating, and has an odor of naphtha or petroleum, a very peculiar taste and an acid reaction.

No. 7.—Grains of solid matter per gallon.............................95.60

Consisting of the following salts:

Sulphate of aluminum (heavy),
Sulphate of lime (faint),
Sulphate of magnesia (faint),
Chloride of iron,
Sulphate of iron,
Free sulphur,
Organic matter.

This is a strong alum water, having a bitter taste and a strong odor of bitumen from the floating oil which is seen upon the surface, and accounts for the organic matter found in the residue. From this oil is derived the tar found upon drying the condensed salts, and which impregnates the soil about the springs.

No. 8.—Grains of solid matter per gallon.............................70.01

Consisting of the following salts:

Sulphate of alumina (strong),
Sulphate of iron (strong),
Sulphate of lime,
Chloride of iron (faint),
Silica (in solution),
Free sulphur,
Organic matter.

No free gases, odor of petroleum, and no traces of the floating oil.

No. 9.—Grains of solid salts per gallon.............................86.58

Consisting of the following salts:

Sulphate of aluminum (heavy),
Sulphate of lime,
Sulphate of magnesia (very faint),
Chloride of iron,
Chloride of potassium,
Chloride of sodium,
Silica (in solution),
Free sulphur,
Organic matter.

This is strictly an alum water, giving quick reactions for alumina. It is remarkably free from carbonic acid and shows no evidence of sulphuretted hydrogen as received. It is the heaviest water of the four acid wells.

No. 10.—Grains of solid matter per gallon..............................9.08

Consisting of the following salts:

Sulphate of lime (faint),
Sulphate of iron (very faint),
Chloride of iron (very faint),
Organic matter.

This is the freest of all the waters from sulphates, excepting No. 1, and has the least solid matter per gallon of any of the springs. Were it not for its traces of organic and tarry matter, it would be remarkably pure water for any purpose.

No. 11.—Grains of solid salts per gallon..............................17.87

Consisting of the following salts:

Sulphate of iron (quite heavy),
Sulphate of lime,
Sulphate of magnesia (faint),
Chloride of iron,
Chloride of potassium,
Chloride of sodium,
Organic matter.

This water much resembles No. 1 in appearance, but has heavier iron and organic acid not found in the former (No. 1).

None of these waters throw out their iron as the oxide, and none of them have floating impurities at all.

No. 12.—Grains of solid matter per gallon..............................71.59

Consisting of the following salts:

Sulphate of alumina (strong),
Sulphate of iron,
Sulphate of lime,
Sulphate of magnesia (faint),
Chloride of iron,
Chloride of lime,
Sulphuretted hydrogen (strong),
Free sulphur,
Organic matter.

This is a sulphur water, with strong odor of sulphuretted hydrogen, giving reactions quickly for alumina, and leaving a tarry residue with acid reaction.

No. 13, or the lake water.—Grains of solid matter per gallon..........25.08

Consisting of the following salts:

Sulphate of alumina,
Sulphate of iron (heavy),
Sulphate of lime,
Sulphate of magnesia (faint),

Chloride of iron,
Chloride of potassium,
Sesquioxide of iron (floating)
Free sulphur,
Organic matter.

The lake water is a clear water, with more or less free sulphur, comprising, as is evident, nearly all the salts contained in the various springs.

Respectfully, REGIS CHAUVENET & BRO.,
Analytical Chemists, St. Louis, Mo.

GALVESTON, TEXAS, September 17, 1898.

ANALYSIS OF MINERAL WATER FOUND AT SOUR LAKE, TEXAS, AT A DEPTH OF 200 FEET.

Gases--
Carbon-oxy-sulphide.
Sulphuretted hydrogen.
Carbonic acid.
Fixed mineral substances--
Silicic acid.
Iron oxide.
Sulphate of lime.
Aluminum oxide.
Sulphate of strontia.
Carbonate of magnesia.
Chloride of sodium.
Iodide of sodium.
Bromide of sodium.
Nitrare of sodium.
Bicarbonate of sodium.

The water is a strong salt salinic mineral water, and its medicinal properties are remarkable. It contains a large amount of iodide of soda and carbonate of magnesia, so that its use in diseases of the intestines and urinary organs is strongly indicated. For rheumatism probably no better remedy than the above water can be found. As it contains carbon-oxy-sulphide and bromides, it will prove beneficial to nervous people.

The water is entirely free of organic matter, which signifies that no products of fermentation are present, which may be injurious to health.

Taken as a whole the water combines in it the healing properties of the waters of Pyrment and Wiesbaden in Germany—certainly a lucky combination. (Signed), F. C. THIELE, Chemist.

It will be seen from the foregoing analysis that the waters of Sour Lake are mainly charged with sulphur, iron and alumina, except that No. 20 is characterized by an entirely different set of ingredients, and a combination nowhere else found in a natural state upon the face of this earth.

No. 6 is the well of "natural lemonade," a glassful of which, with a small quantity of sugar, makes a delicious summer beverage.

But it is more particularly to the medical properties of this rare collection of waters that the writer wishes to call the attention of readers.

It is not asserting too much to state emphatically that Sour Lake water has no superior on earth, so far as known and published, as a remedy for disturbances of digestion. No matter in what stage—*dyspepsia, indigestion and gastric catarrh* will yield to the beneficial influence of this water.

A wide-spread knowledge of this fact should make it one of the most popular natural remedies in the country, and thousands are living to-day who can readily attest the correctness of the statement.

Besides its specific nature for the tripod of ailments just mentioned, and which alone should mark it as one of the most valuable waters known, its effect upon the blood in removing impurities therefrom is no less characteristic and prompt. Likewise are pimples, blotches, boils, and other blemishes of the skin removed.

Besides the foregoing, a large variety of other ailments are either cured or markedly relieved by the internal use of Sour Lake water.

I have seen malarial fever cured in a few weeks without the use of a single grain of medicine beyond that found in the water from these wells, and this experience of mine has been corroborated by that of others. This property of the water, therefore, becomes extremely valuable when we consider the great number of people both in the northern and southern portion of our country who are the victims of malaria. This remedial nature of the water might be reasonably inferred, however, from the well-known effect of sulphur preparations upon parasitic diseases.

Hepatic torpor, biliousness and jaundice even of very obstinate type have time and again been relieved by a few weeks' stay at Sour Lake.

A few years ago, a remarkable case of dropsy,—general anasarca—was cured by the use of this water, and the cure remains up to the present date. But the writer is unable to ascertain whether this dropsy was of cardiac, renal or hepatic origin. It is claimed that Sour Lake water is beneficial in Bright's disease, but this opinion is *sub judice* at present, and will be thoroughly looked into this summer when the resort will be thrown open for the reception of guests.

Besides the wells at Sour Lake, there are bathing pools found there which are as remarkable for their particular virtues as the former are for curing the diseases just alluded to.

For cutaneous troubles there are no baths found anywhere so certain in their effects as these. For eczema in particular, no baths in the world are better than the mud baths of Sour Lake. Besides eczema, all the itchy and scaly diseases that flesh is heir to find prompt and certain relief in their employment.

A single case will serve to illustrate this point. John L., aged forty-two years, a gentleman residing in Galveston, was attacked in January, 1898, by a most obstinate case of eczema. It involved his face, hands, and feet, and great masses of crusts would form and fall from these portions of his body, while the characteristic ichor which alternately oozed from these parts rendered him an object of general commiseration. In the treatment of his case, I employed all the preparations I had ever used, including astringent salves and washes, exclusion of light, etc., but to no avail. At times he improved to such a degree that his cure was predicted in a day or two, but he invariably relapsed when the time came for discharge, and these relapses occurring so often, after six weeks, I directed him to Sour Lake, in order to avail himself of the waters concerning which I had heard so much.

Mr. L. spent one month at Sour Lake, fairly living in the acid mud baths much of the time, and receiving benefit from the start.

He returned to this city at the end of that time cured, and has remained cured up to the present day.

This is a sample of perhaps thousands who could boast of the same experience. These baths appear to be a prompt and pleasant specific for all such cases, and any one afflicted with eczema or any of its relations, can confidently rely upon a cure by testing them.

### LOCATION, GENERAL INFORMATION, ETC.

Sour Lake is in Southeast Texas, about twenty miles northwest of Beaumont, and eight miles from Sour Lake Station on the Southern Pacific Railroad. Between these last two points conveyances are run to meet the trains and accommodate the visitors, and the managers of the resort are zealous in their endeavors to please their patrons.

Aside from the medicinal character of Sour Lake, a world of sport awaits the visitor if he chooses to alternate his treatment there with more exciting pastime. The "Big Thicket" alluded to in the opening of this article, and near at hand, is one of the most noted hunting places in the South. Here deer, turkeys, squirrels, bear, and even wilder game abound in large quantities, and those whose taste inclines them in the direction of exhilarating and exciting sport can indulge in hunting to their heart's content.

Quite recently this locality has developed signs of oil, and flowing wells of this commodity are to be found on and around the grounds. With tar and bitumen oozing from the surface near the lake, and commingling with its waters, this development might have been expected.

Thus the health-seeker at Sour Lake can find abundant sources of pleasure and recreation while undergoing his cure. This item of diversion is invaluable to the invalid, who too often finds dullness and lonesomeness attendant upon his stay at the usual watering-place.

